# Impact of Coping Skills Training on the Quality of Life Among the Daughters of Mothers with Breast Cancer

**DOI:** 10.30476/ijcbnm.2020.83048.1132

**Published:** 2020-10

**Authors:** Sedigheh Khanjari, Mina Mianji, Mitra Hakim Shooshtari, Hamid Haghani

**Affiliations:** 1 Nusing Care Research Center, Department of Pediatrics, School of Nursing and Midwifery, Iran University of Medical Sciences, Tehran, Iran; 2 Department of Pediatrics, School of Nursing and Midwifery, Iran University of Medical Sciences, Tehran, Iran; 3 Mental Health Research Center, Tehran Institute of Psychiatry, School of Medicine, Iran University of Medical Sciences, Tehran, Iran; 4 Biostatistics, School of Management and Information Technology, Iran university of Medical Sciences, Tehran, Iran

**Keywords:** Adolescent, Breast cancer, Coping skills, Mother, Quality of life

## Abstract

**Background:**

Cancer affects the quality of life (QoL) of patients and their families. The purpose of this study was to determine the effect of coping skills training on the QoL among daughters of mothers with breast cancer.

**Methods:**

In this quasi-experimental pre-test/post-test design, data were collected from 70 participants (35 in each of the control and education groups) from January 2016 to July 2017 in Imam Khomeini and Rasole-e-Akram Hospitals in Tehran. The education group participated in a workshop and group discussion (groups of 5 to 8 participants) with the presence of a pediatric psychiatrist and two pediatric nurses, and then a follow up program was performed. The Pediatric Quality of Life Inventory version 4.0 was used in this study in two stages of pre-test (before education) and post-test (four weeks later). Data were analyzed through SPSS, version 21 using independent t-test and paired t-test for comparison of the mean scores of the two groups, with the significance level of 0.05.

**Results:**

After the education, there were significantly improved scores of the QoL in the dimensions of physical functioning (P<0.001), emotional functioning (P<0.001), and school functioning (P<0.001) in the study group compared to the control group. The social functioning did not show a significant change (P<0.083).

**Conclusion:**

The findings of the study confirm that coping skills training can lead to the improvement of QoL in adolescent daughters of mothers with breast cancer. Healthcare professionals must provide the mothers and daughters with information about breast cancer and instruments to handle their situation to promote the daughters’ QoL.

## INTRODUCTION

The occurrence of cancer in parents is associated with various complications for family members, especially children, which can lead to a decline in their cognitive, emotional and social functioning. ^1^ Breast cancer is the most common cancer in women worldwide and the leading cause of cancer death in the world. ^2^ In Iran, breast cancer is ranked the first among all cancers diagnosed in women, and its incidence is increasing. ^3 , 4^

The occurrence of cancer in mothers can cause many mental and emotional problems in adolescents’ life. Diagnosis of breast cancer, the ensuing side effects, and bodily changes after treatment can lead to long term distress and increased tension in family members. ^5^ Furthermore, female adolescents are at more risk ^6 , 7^ since they are at the puberty stage and avoid using coping strategies. ^8^ Adolescent daughters of mothers with breast cancer may be afraid of developing breast cancer in the future which can lead to distress and anxiety throughout adolescence, especially the first year after a parent is diagnosed with cancer. ^5 , 9 , 10^

Cancer affects the quality of life (QoL) of an afflicted person, their family members, and disrupts other familial structures. ^11 - 14^ Six months after diagnosis of breast cancer, regardless of improved QoL, results of a study in Iran reported the family caregivers’ struggle in coping with the situation. ^15^ Results of studies reveal increased anxiety and psychological distress in children living with parental cancer. ^16 , 17^ Adolescents may use unhealthy coping mechanisms that include avoidance of any communication surrounding the disease, which may particularly demonstrate behavior problems when a parent has cancer. ^18 , 19^ Adolescents try to receive information and talk with peers who are going through similar situations. ^20^ Special attention must be paid to the emotional and psychological needs of the family caregivers of women diagnosed with breast cancer in order to promote their QoL. ^20 , 21^ Holding interventional programs is recommended with training in a wide range of coping skills in cases of family caregivers with cancer. ^9 , 10 , 11 ,^

Taken together, results of some studies indicate that some family caregivers have more problems with adjusting to the situation, which may finally lead to lower QoL. Therefore, it is important for adolescents to be able to accept their parents’ condition. That is why incorporating QoL assessment in combination with coping strategies in clinical settings that are currently accepting patients and family caregivers is essential for supportive interventions. ^21 - 23^ On the other hand, research shows that the mother and daughter’s behaviors are influenced by culture. Different expectations of behavior in mothers and daughters depend on their ethnicity; thus, studies should be carried out in different countries, ^24^ especially for female family caregivers. ^25^ Therefore, this study was conducted with the aim of determining the impact of coping skills training on the QoL among daughters of mothers with breast cancer.

## MATERIALS AND METHODS

This quasi-experimental pre-test post-test design was conducted from January 2016 to July 2017 for a group of adolescents aged 15 to 18 with mothers suffering from breast cancer that referred to educational and referral hospitals of Imam Khomeini (education group) and Hazrat-e Rasool (control group) in Tehran. Initially, the researchers (SH and MM) contacted the nurses working in the oncology and chemotherapy units where women with breast cancer were being treated. The goal of the study was explained to the nurses, so that they can assist and introduce it to the mothers. In the next step, the researcher (MM) visited the eligible mothers and explained the goal of the study and asked for their consent to contact their adolescents. The adolescent daughters were then informed about the study, and written consent was obtained from those who volunteered. Initially, the adolescent daughters completed the questionnaires at the hospital. Four weeks after the study, the participants received the questionnaires via regular mail that included a prepaid envelope for return. 

Two hospitals, i.e. Imam Khomeini Hospital Complex as the largest center for women with breast cancer in the country and Rasoul-e-Akram Medical Research Center as the most important center for Iranian breast cancer patients were selected. Each is affiliated to one of the city’s medical universities in Tehran. The inclusion criteria for the mothers were having been diagnosed with breast cancer, being under chemotherapy, and having an adolescent daughter. The inclusion criteria for the adolescents were not having a history of or current psychiatric or neuropsychological disorders and not having been diagnosed with cancer of any type. The exclusion criteria consisted of adolescents who did not participate in all educational sessions or follow-up sessions, stressful events, and attendance in specific educational training related to QoL throughout the education and follow-up periods.

To determine the required sample size with 95% confidence and 80% power, the sample size formula in each group of 35 subjects was calculated assuming that the effect of training in coping skills on the QoL of adolescents of mothers with breast cancer was at least d=9 in comparison with the control group. That would ensure that the impact of the educational program would be statistically meaningful. The standard deviation of the Pediatric Quality of Life Inventory (PedsQL) in each group was estimated to be 13.5. ^26^

z_0.975_=1.96

z_0.8_=0.84

$n=\frac{{\left(\mathrm{1.96}+\mathrm{0.84}\right)}^{2}(13.5^{2}+13.5^{2})}{9^{2}}35$

After referring to the chemotherapy and radiotherapy departments of each hospital and reviewing the archived records (over the past year), the researcher (MM) selected 342 women with breast cancer. One hundred twenty one of them who had adolescent daughters aged 15-18 years old were contacted and enrolled in the study, while 46 did not agree that their adolescents participate, leaving a total sample of 75 mothers. The researcher then explained the goal of the study to them in detail before inviting them to participate in the study. Finally, 75 women (37 in the control group and 38 in the education group) agreed to participate in this study by completing the consent form. Of these, in the education group, two daughters did not return the questionnaires at follow-up and one daughter declined further participation. In the control group, two participants reported that they discontinued participation in the study (being busy with work and family). Thus, the final sample consisted of 70 participants that completed the questionnaires at pre-test and post-test (35 in the education group, 35 in the control group). Additionally, medical records were used to complete the questionnaire. All questionnaires were distributed and collected by the researcher (MM).

The data collection tools included the socio-demographic information questionnaire. This questionnaire includes adolescents’ information such as age, education level and birth order, and parents’ information such as age, surgery status, education level and number of children.

PedsQL Version 4.0 was originally developed in the English language by Varni et al. in 1998. The questionnaire assesses the QoL in four aspects of physical, emotional, social, and academic education through 23 items where each item is given a point based on the five-point Likert scale. Scores range from 0 to 4, with 0 meaning never having a problem; 1 meaning almost never having a problem; 2 indicating sometimes having a problem; 3 indicating often having a problem; and 4 standing for almost always having a problem. Items are reversed-scored and linearly transformed to a 0-100 scale (0=100, 1=75, 2=50, 3=25, 4=0), so that higher scores indicate better functioning. ^27^

The instrument had internal consistency reliability for the total scale score (α=0.88), Physical Health Summary Score (α=0.80) and Psychosocial Health Summary Score (α=0.83). ^27^ The validity was demonstrated through the known-groups method and factor analysis with good standards. Comparison of the samples with chronically ill, acutely ill, and healthy children for all scales has confirmed the differences among the three groups using One-Way ANOVA’s. It was verified that healthy children showed higher scores than ill children. Most self-report scales and proxy-report scales surpassed the reliability standard of 0.70. ^28^ The mean score for physical functioning was achieved through the scores obtained from the subjects’ response to eight questions including those on the patterns of running, walking more than one block, carrying heavy objects, bathing, having injury or pain and low energy levels, doing chores around the house, and participating in sports activities or exercise ranging from 0 to 32 points in total. The mean score for emotional functioning was achieved through the score obtained from the subjects’ response to five questions, including those about feeling of fear and apprehension, feeling nostalgic or depressed, feeling anger, communicating while asleep, and worrying about what will happen, ranging from 0 and 20 points in total. Social functioning is achieved through the score obtained from the subjects’ response to five questions, including those on communication, being abused by other adolescents, and tolerance when playing with peers, being rejected by other adolescents, being bothered by other adolescents, which ranges between 0 and 20 points. The school functioning is obtained through the score obtained from the subjects’ responses to five questions, which include questions on attention span in class, forgetfulness, classroom assignments, and absenteeism, not feeling good about going to school, ranging from 0 and 20 points. In Iran, the PedsQL has been validated by three experienced psychologists and reviewed by the developer. The reliability of the questionnaire used in this study was Cronbach’s alpha (α=0. 84) and its content validity index in PedsQL was 84%. The four subscales of physical functioning, emotional functioning, social functioning, and school functioning were reported to be 80%, 86%, 83% and 88%, respectively. ^26^

This study was approved by the ethics committee of Iran University of Medical Sciences (Grant no. IR.IUMS.REC 94-03-123-2677) and the researchers abided by the Helsinki Declaration. The mothers provided informed verbal and written consent. Mothers and their adolescents participating in the study were reassured that their failure to complete their participation would not prevent them from receiving the routine treatment, and that the adolescents were allowed to enter the study and withdraw at any stage of the trial. The researcher also underlined the confidentiality of information obtained from them throughout the process. 

The education group received the training sessions as follows: the first three sessions lasted one hour and forty-five minutes and the last session continued for two hours (based on the decision of the research team) by a child and adolescent psychiatrist and two pediatric nursing professionals (authors) in the hospital. The training included group discussions with five to six participants. The training included a workshop, simulation, and follow-up program, designed to help improve the education of the adolescents. The workshop was conducted in two 6-h sessions to cover all female adolescents. Twenty-two adolescents participated in the first workshop, and 16 in the second. The first workshop consisted of 4 groups of 5 to 6 adolescents. The second workshop consisted of three groups of 5 to 6 adolescents. The contents were described to the adolescents using lecture, PowerPoint slides, small group discussion, question and answer, interactive discussions, practice with moulage, educational film, and finally practical performance by the participants. The same educators were responsible for running the educational program in two workshops.

Of those four training sessions, the first was spent on getting acquainted with the female adolescents and their problems. The following sessions began with reviewing the contents of the previous sessions followed by a new discussion involving the daughters and encouraging group members to share experiences and make connections with one another. The topics addressed in the sessions included diagnosis and treatment consequences, tensions, feelings of guilt, communication, rise of responsibilities, changes in different aspects of life, behavioral changes, fears, sighting the parents’ weakness, loss of normal life and activities, and emotional support from partner and family relationships. The education details are described in Table 1. 

**Table 1:** The Contents of the Sessions

Sessions	Objectives	Summary of Educational Content	Time (hours)
1	Getting familiarized with learners, cancer description, how cancer spreads, cancer staging	Getting to know and analyze learners’ knowledge about the concept of cancer	1.45
		Description of cancer, Cancer staging and risk factors for cancer	
		Group discussion and sharing experiences	
		Question and answer	
2	Adaptation mechanisms, active adaptation, avoidance adaptation. Internal and external reactions	Description of emotions provoked in mothers and adolescents, including (fear, loneliness, hopefulness, anxiety, anger, sadness)	1.45
		Explaining changes made in the family (including routines and responsibilities, and providing recommended methods (communicating, maintaining family and friends circles)	
		Providing coping strategies and adaptation mechanisms	
		Group discussion and sharing experiences	
3	Effective factors in cancer, breast self-examination, cancer treatment and conclusion	Explaining the effective factors of cancer: aging, lack of mobility and exercise, smoking, inadequate consumption of fruits and vegetables, yellow- or dark-leaf vegetables	1.45
		Explaining self-care and self-examination of breast with related images	
		Explaining all types of breast cancer and respective treatments	
4	Supporting resources, supporting and delivering different coping strategies in dealing with stress	Describing sources of support: parents, counselors, nurses, doctors, social workers, the Internet or support groups	2
		Describing conditions after treatment	
		Group discussion and sharing experiences and conclusion	

The material was developed by a literature review and our previous experiences on coping strategies in family caregivers of women with breast cancer. ^15 , 21^ In this study, all the materials were approved by four experts including a child and adolescent psychiatrist, two pediatric nursing professionals, and one psychiatric nursing professional. The end of the session included a booklet about each training section and related teaching materials. This booklet, published by the National Cancer Institute entitled, “When Your Parent Has Cancer: A Guide for Teens” ^29^ was translated into Persian. This book was assessed for translation and cultural adaptation by five Iranian experts. In addition, a breast cancer self-exam for the adolescents’ daughters and breast cancer prevention points were also addressed and covered. The information and training that would be given to the daughters consisted of topics on how to do breast self-exam through PowerPoint presentation and moulage; the researcher gave a booklet about breast self-exam to the daughters at the end of the session.

The control group received only educational pamphlets and posters in the section on illness and chemotherapy care. The control group was provided with the booklet after the completion of the study. In this study, adolescents were followed up on a weekly basis with questionnaires on educational materials via telephone and social networks during the interval between pre-test and post-test periods. They also asked questions about the contents of the booklet, while their questions were answered and guided. The researcher asked about changes at home and how to cope with the changes were reminiscent of support sources including counselors and assistants, changes in their feelings and their mothers. Furthermore, adolescents asked questions about breast self-examination and how to prevent it and were referred to a specialist, if needed. 

The data were analyzed in SPSS software version 21, using statistical tests including Chi-square test, independent t-test, paired t-test and analysis of variance, with the significance level of P<0.05. A clinically significant change in the QoL of family caregivers was estimated by calculating the effect size. The effect size estimation was calculated by subtracting the mean of one group from the other and dividing the result by the standard deviation of the same score at the pre-test. According to Cohen, an effect size of 0.20 is considered small, greater than 0.50 is considered moderate, and greater than 0.80 is deemed large. ^30^

## RESULTS

To evaluate the distribution of the variables, the results showed that the outcome variables were all normally distributed. The dropout rates in the education (n=3) and control (n=2) groups were 10% and 7%, respectively, due to cancellation, lack of data, or failure to return the questionnaire. The demographic characteristics of the adolescents and the mothers are summarized in Table 2. The mean age of the adolescents in the education and control group was 16.74±1.47 years and 17.17±1.42 years, respectively. The mean age of the mothers was 43.31±6.12 years in the education group and 44.43±6.49 years in the control group. The education level of more than half 19 (54%) of the mothers in this study was diploma and 17 (49%) mastectomy (was observed in the education group) (Table 2). The findings shown in Table 2 indicate that there were significant differences between the two groups of adolescents in terms of education (P=0.01), birth order (P<0.001), and parents’ number of children (P=0.001), but the results of analysis of variance on non-homogeneous variables indicated that there was no significant difference between the two groups (P=0.06, P=0.0 78 and P=0.06, respectively). It should be noted that the analysis of variance showed that QoL scores in different subgroups of variables education, birth order in children, and the number of children were not statistically different between the education and control groups. 

**Table 2:** Descriptive characteristics of the adolescents and their mothers with breast cancer in the education (n=35) and control (n=35) groups

Variables	Education group N (%)	Control group N (%)	P value*
Children			
Birth order			<0.00
1	1 (2.9)	16 (45.7)	
2	22 (62.8)	7 (20)	
3	12 (34.3)	5 (14.3)	
4	0 (0)	7 (20)	
Level of education			
Secondary-high school			0.01
First	1 (2.8)	5 (14.3)	
Second	12 (34.3)	18 (51.4)	
Third	15 (42.9)	4 (11.4)	
Fourth	7 (20)	8 (22.9)	
Mothers			
Surgery status			0.15
Breast removal	17 (49)	23 (66)	
Remain	18 (51)	12 (34)	
Level of education			0.79
Primary school	1 (3)	0 (0)	
Secondary school	5 (15)	4 (11)	
Diploma	19 (54)	18 (52)	
University	10 (28)	13 (37)	
Number of children (%)			0.001
1	0 (0)	14 (40)	
2	18 (52)	6 (17)	
3	15 (43)	8 (23)	
4	2 (5)	7 (20)

* Chi-Square test

The findings displayed in Table 3 show that there is no significant difference in the overall QoL and any of its factors prior to the intervention between education and control groups. This difference was significant and the mean score was higher in the education group after the intervention for emotional (P=0.003), social (P=0.004) and school functioning (P=0.004), and overall score of QoL (P<0.001) except for physical functioning (P=0.19) (Table 3). The adolescents scored significantly higher overall on the overall score of QoL (P<0.001) and physical (P<0.001), emotional (P<0.001) and school functioning (P<0.001) after the intervention, except for social functioning in the educational group. 

**Table 3:** Comparisons of means, Standard Deviations and Effect size of all aspects of the quality of life in adolescents of mothers with breast cancer in education and control groups

Variables	Groups	Pre test Mean±SD	Post test Mean±SD	P value** (within)	Effect size
Physical Functioning	Education	68.6±12.4	74.8±9.1	<0.001	-0.50
	Control	69.73±9.6	72.0±8.4	<0.001	-0.24
	P value* (between)	0.663	0.192		
Emotional Functioning	Education	57.3±16.1	65.6±11.9	<0.001	-0.52
	Control	60.7±12.3	56.3±13.1	<0.001	0.36
	P value* (between)	0.322	0.003		
Social Functioning	Education	78.3±17.5	80.4±16.1	0.083	-0.12
	Control	74.1±17.5	68.7±17.1	0.001	0.31
	P value* (between)	0.326	0.004		
School Functioning	Education	73.6±16.2	79.9±14.2	<0.001	-0.39		
	Control	73.4±14.4	70±13.2	0.004	0.24
	P value* (between)	0.969	0.004	<0.001	
Quality of life	Education	69.3±12.0	75.1±9.5	-0.48
	Control	69.5±9.1	67.4±8.8	<0.001	0.23
	P value* (between)	0.932	<0.001		

* t-test;

** Paired t-test

## DISCUSSION

The results of this study showed that life with mothers diagnosed with breast cancer had a significant impact on all aspects of QoL among adolescents. These results are compatible with a study which stated that such adolescents and their families faced a myriad of problems due to the nature of the disease, ^31^ highlighting the need for emotional support for such adolescents. 

One way to help the affected adolescents is to train them in coping skills. According to the results of other studies, interventional programs are significantly helpful to family members by improving communication and coping skills. They are also important in reducing anxiety and increasing the QoL of family caregivers. ^24 , 32 , 33^ In the current study, interventional programs led to an increase in the score of QoL among female adolescents of mothers with breast cancer. 

In the present study, coping skills training had the most positive impact on the emotional functioning of QoL. Adolescent daughters of mothers with breast cancer experience a range of feelings about their mothers’ condition. They may feel apprehensive about developing breast cancer themselves in the future. ^5^ Informing the child about what has happened while considering the child’s age and developmental phase can help them adapt to the parent’s illness and make them feel secure and at peace. ^6^

The results of this study demonstrate that coping skills training can increase the QoL in physical functioning in the education group as well. In some cases, adolescents try to take on additional domestic responsibilities. By creating changes in daily routines and everyday tasks, the role of each family member changes. New responsibilities include medicine intake and treatment schedules, which are indicative of changes in family responsibilities. Lack of time and energy for social interactions, and concerns about academic performance are the other daily stressors that can create issues regarding physical discomfort as well. ^33^ However, the difference was not significant in the physical functioning dimension of the QoL between the control and education groups after the intervention; this could be due to the sensitivity of the adolescents in the control group after reading the PedsQL. They have probably thought about their activity and its amount. 

The results of the current study also indicate an increase in the school functioning of the QoL subscale after the intervention. Adolescents of parents with cancer are not able to concentrate and complete school assignments and experience a decline in school performance. ^10^ Furthermore, these adolescents have more emotional problems and stress response symptoms than their peers in the control group who do not have cancer parents or mothers. ^34^

Our findings in this study showed increased mean score, but not significant in the social functioning of adolescents after the intervention in the education group. In general, the relatively high mean score before the intervention in the education group may affect the possibility of a significant improvement in the score after the intervention, depending on ceiling effects. Sociological theories emphasize that culture has a significant impact on the approach, performance and adaptability of families against stress. ^35^ In many cultures, adolescents may receive emotional support from more distant members of the family, friends or society, especially spiritual websites and forums. This kind of social support allows the adolescents to discuss their negative feelings and anxieties in a comfortable and safe environment. ^10^ Findings of a study emphasize that friends continue their friendship with an adolescent child of a parent with cancer normally as before. ^36^ In a study, children who were emotionally protected by friends and family showed lower degrees of depression. ^37^ In another study, adolescents noted the importance of communicating with their siblings, and said they felt better sharing the same experience, which helped them to accept and create emotional balance. ^38^ Social support from mothers for their children could be a major contributor to child psychological adaptation. Also, having better relationships with family members and the level of mother’s adaptation and creativity in managing the situation plays an important part in children’s coming to terms with the new circumstances. ^39^

Abundant information on the disease, its side effects, treatment, and potential behavioral changes in their parents can help reduce anxiety in adolescents aged 14-18 years old. ^40^ Therefore, supportive educational programs are suggested. In this study, the use of coping skills training within a group was considered as an effective learning method during which adolescents could acquire and use effective coping skills. As reported in in this study, group discussions were used in adolescent education. According to researchers, this method can reduce stress by creating the opportunity for the participants to share their experiences and learn about the hardships they have in common.

In the second step, QoL in the control group showed statistically significant changes, which is indicative of worse QoL than pre-intervention. It is probable that the participating adolescents realized the importance of the issue after studying the questionnaire and pamphlet. However, provoking a sense of curiosity toward cancer and its ensuing problems may also lead to some degree of stress and internal conflict.

This study had several limitations. Firstly, this study did not reflect the interaction of possible influencing variables such as the mother’s QoL and psychological functioning. A further limitation was the short follow-up time. In most cases, parents were afraid of increasing their adolescents’ extent of knowledge on their condition (which could lead to increased distress) and, therefore, were not willing to participate in the study. Considering the fact that in this study the results cannot be generalized to other situations, (i.e. to fathers with breast cancer or male adolescents of mothers with breast cancer), it is suggested that a similar but generalizable study be conducted on the effects of coping skills training. 

## CONCLUSION

The use of this type of education could be a part of community-based nursing practice and, therefore, the knowledge of breast cancer and its prevention could be increased in the society through effective training programs. The study findings confirmed the effectiveness of coping skills training on the QoL of adolescents. It is one of the responsibilities of the nurses to provide psychological support to daughters and boost their knowledge on their mothers’ disease to help them better adapt to the new condition. This program is, therefore, recommended to be implemented at the primary preventive level in community health centers. It is also recommended that the long-term effects of coping skills training should be examined. Given that the social dimension score failed to show a significant difference after the intervention in this research, it is suggested that effective coping skills should be developed for adolescents in their interpersonal relationships and social support.
